# Design and Evaluation of Using Head-Mounted Virtual Reality for Learning Clinical Procedures: Mixed Methods Study

**DOI:** 10.2196/46398

**Published:** 2023-08-30

**Authors:** Siew Tiang Lau, Rosalind Chiew Jiat Siah, Khairul Dzakirin Bin Rusli, Wen Liang Loh, John Yin Gwee Yap, Emily Ang, Fui Ping Lim, Sok Ying Liaw

**Affiliations:** 1 Alice Centre for Nursing Studies Yong Loo Lin School of Medicine National University of Singapore Singapore Singapore

**Keywords:** user experience, acceptability, usability, virtual patient, clinical procedure, immersive, nursing student, virtual reality, education, performance

## Abstract

**Background:**

The capacity of health care professionals to perform clinical procedures safely and competently is crucial as it will directly impact patients’ outcomes. Given the ability of head-mounted virtual reality to simulate the authentic clinical environment, this platform should be suitable for nurses to refine their clinical skills for knowledge and skills acquisition. However, research on head-mounted virtual reality in learning clinical procedures is limited.

**Objective:**

The objectives of this study were (1) to describe the design of a head-mounted virtual reality system and evaluate it for education on clinical procedures for nursing students and (2) to explore the experience of nursing students using head-mounted virtual reality for learning clinical procedures and the usability of the system.

**Methods:**

This usability study used a mixed method approach. The stages included developing 3D models of the necessary instruments and materials used in intravenous therapy and subcutaneous injection procedures performed by nurses, followed by developing the procedures using the Unreal Engine (Epic Games). Questionnaires on the perception of continuance intention and the System Usability Scale were used along with open-ended questions.

**Results:**

Twenty-nine nursing students took part in this questionnaire study after experiencing the immersive virtual reality (IVR) intervention. Participants reported largely favorable game perception and learning experience. Mean perception scores ranged from 3.21 to 4.38 of a maximum score of 5, while the mean system usability score was 53.53 of 100. The majority found that the IVR experience was engaging, and they were immersed in the game. The challenges encountered included unfamiliarity with the new learning format; technological constraints, such as using hand controllers; and physical discomfort.

**Conclusions:**

The conception of IVR for learning clinical procedures through deliberate practice to enhance nurses’ knowledge and skills is promising. However, refinement of the prototypes is required to improve user experience and learning. Future research can explore other ways to use IVR for better education and health care purposes.

## Introduction

### Overview

Generation Z students are well recognized to have different learning needs and preferences than previous generations [1]. With the advancement of medical technology and treatment, coupled with dynamic clinical settings, the ability of nurses to perform clinical procedures safely and competently is of paramount importance. The issue of ensuring competency prior to performance on actual patients remains crucial for patient safety. Therefore, new teaching strategies are needed to bridge the teaching-learning gap to create an effective learning environment. The use of technology to meet the challenges of medical education has been well documented [2,3]. Simulation sessions using manikins in clinical laboratory settings have been established for more practice. However, these simulation sessions are usually conducted in small groups and are labor and resource intensive. Studies also suggest that these conventional teaching methods do not fully meet the needs, interests, and learning preferences of Generation Z students [4,5]. The novel discovery of virtual technology has been explored for its effectiveness in enhancing students’ learning in nursing education [3,6,7].

Immersive virtual reality (IVR) is highly visual and conducive to procedure-focused learning. IVR provides first-person active learning where learners can interact with 3D objects and characters [8]. With immersive 3D virtual reality (VR), learners can perform clinical procedures in a real clinical environment. It is well established that IVR as a medium of instruction facilitates interaction and immersion [9]. This enhances learning through the learner’s psychological constructs, such as presence and agency. Presence forms the conditions, and agency gives autonomy of control to gain experience through immersive technology [9]. In designing IVR training, it is important to understand the factors influencing learning. Factors such as perceived realism, usability, and learning are crucial to learners’ attitude toward the use of VR [10]. Hence, VR features, learning experiences, usability, and learning theories that guide the learning process are vital constructs when designing an IVR training environment [10].

The transformation of nursing education has been rapidly evolving with the advancement of VR technology. IVR allows learners to obtain repeated hands-on practice to understand concepts and master procedures, exercise their problem-solving skills, and develop confidence prior to managing actual patients in clinical settings. IVR allows learners to practice safely without risk to patients and empowers them for self-directed and regulated learning. IVR was reported to be used frequently to teach procedural-practical knowledge [11]. Although there have been nursing VR simulations conducted to improve technical knowledge and proficiencies, these studies focused mainly on linear step-by-step intervention, and the order of the steps played an important role [12,13]. Systematic reviews evaluating the effectiveness of IVR report that it improved knowledge and learning experiences, but the reviews’ findings were inconclusive for skills, satisfaction, confidence, and performance in health care education [14-17].

### Theoretical Framework

The conceptual frameworks that guided this study are cognitivism, constructivism, and experiential learning through deliberate practice [18]. Cognitivism theory has been used for information processing in the context of VR. Learning in VR involves immersion, interaction, imagination, motivation, and transformation of new knowledge to enhance problem-solving capability [18,19]. Through the constructivism approach, students engage with the environment and are actively involved in building a scaffold for their learning to construct and develop new skills and meaningful knowledge [20,21]. Experiential learning refers to knowledge creation through the acquisition and transformation of experiences [22]. Through first-hand individual experiences using IVR, students are motivated to learn more in a safe and convenient environment for practice. The interaction with a task in VR allows the construction of learning experiences via abstract, active, and reflective processes of the experiential learning cycle [21,23]. With experiential learning and timely built-in feedback, students will be able to work on areas that they need to improve through self-analysis and reflection and achieve competence through meaningful learning.

**Figure 1:**
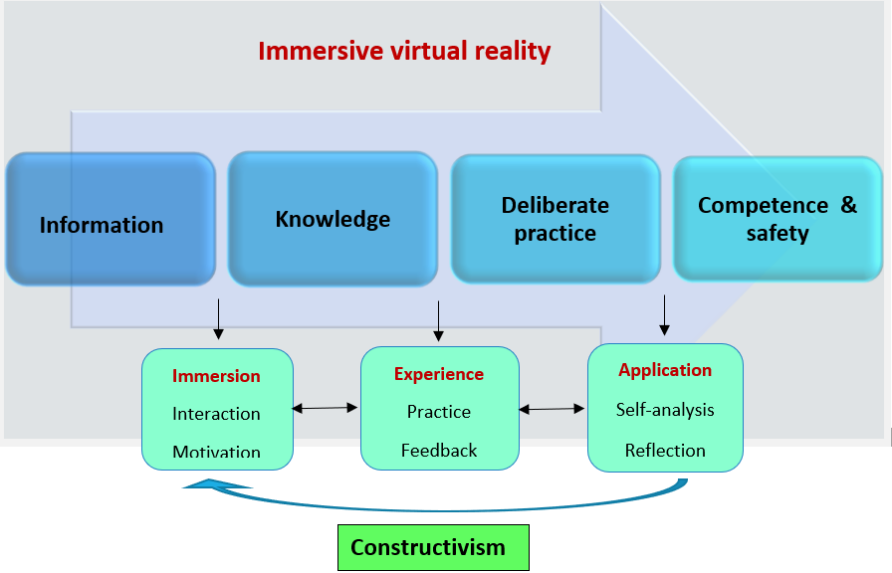
Conceptual framework for learning clinical procedure using virtual reality.

Deliberate practice is the repetitive performance of a cognitive or psychomotor task in a focused domain combined with rigorous skills assessment [24]. Through deliberate practice, students learn new knowledge, analyze, reflect, and problem-solve cyclically for self-improvement [22,25]. With mastery of their experience, students can use deliberate practice, receive feedback on their own performance, improve self-efficacy, and construct new knowledge through experience, as shown in Figure 1.

The aims of this study were (1) to describe the design of a head-mounted virtual reality system and evaluate it for education on clinical procedures for nursing students and (2) to explore the experience of nursing students with head-mounted VR for learning clinical procedures and the usability of the system.

## Methods

### Design and Implementation of IVR Clinical Procedures

#### Design and Development

##### Overview

In this study, 4 key elements were considered when designing the VR prototype to create an engaging and immersive experience for users. The first element was accessibility and inclusivity for all learners and involved software components, adjustable height, and visual and audio design to minimize cybersickness. The second element was locomotion and navigation to allow the learners to navigate the virtual space intuitively. The third element was visual fidelity; the graphics were designed in a detailed and realistic way to provide good immersion for the IVR experience. The fourth element was motion tracking, where the learner’s head and hand movements were detected to allow real-time interaction. An additional consideration was the degrees of freedom and interactivity whereby the learner was given control of their learning [9].

A real-time 3D creation tool, Unreal Engine 5 (Epic Games), was used to support the development of the IVR procedures, which reinforced the real-time interaction of the learner, clinical instructor, and patient in a virtual ward [26]. The design and development of the IVR clinical procedures were a close collaborative effort among the nursing school, health care institution, and IT experts in a systematic process. First, the faculty and nurse clinicians discussed and created clinical scenarios and consolidated common errors, critical steps, and safety issues pertinent to each procedure during the developmental stage.

**Figure 2:**
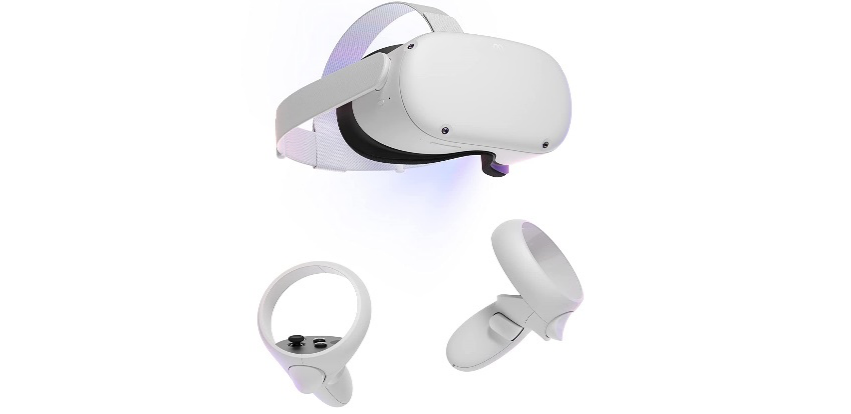
Oculus Quest (with 2 hand-held controllers).

Then, students accessed the proposed immersive simulation of the clinical procedures by wearing a 3D VR headset with 2 handheld controllers (Oculus Quest 2; Reality Labs), shown in Figure 2. Using the Oculus Quest, each student could visually experience a typical clinical session modeled after a real laboratory with its procedures, which they might have experienced before in their typical laboratories. The simulation was highly interactive, leveraging the handheld controllers for the left and right hands to enable and engage the students in executing the same simulated procedures vividly seen in their facilitated laboratory demonstrations. With the rich visual media and interactive experience, students could practice the simulated virtual procedures in their own time and space without mental stress, external distractions, fear of hazardous consequences, or creation of any clinical waste.

Within the IVR program, 3 stages for playing using the scaffold learning approach, namely, the orientation, practice, and assessment modes, were developed.

##### Orientation

**Figure 3:**
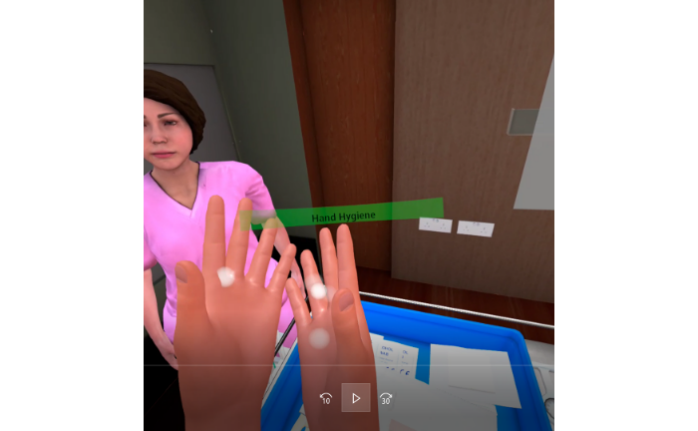
Hand hygiene.

New users were guided by a tutor and an instructional video on the features and safety aspects of using the IVR clinical procedures for learning. When they put on the Oculus Quest 2 for the first time, they were required to complete virtual tutorials on how to maneuver the controllers, how to perform handwashing by rubbing 2 hands together using the controllers (Figure 3) and putting on and removing gloves to optimize their gaming experience.

#### Practice Mode

Visual cues were provided when the learners learned how to proceed from one step to the next. With the built-in checklist, ample opportunities were given for the learners to learn from their experiences. With timely feedback based on the student’s responses, they could rationalize their actions and reactions and thus decide the next course of action or repeat the session. Finally, after the learners gained confidence through practice, made sense of the procedures, and built on their prior learning, they were ready for assessment. No cues were given, and the students could see their results and repeat the practice when required. This approach aimed to allow the construction of knowledge through experience and deliberate practice. It boosted self-regulated learning, as learners could adjust their learning according to their own pace to either move to the next level or remain in the practice mode until they were ready for assessment [27].

##### Assessment Mode

No cues were provided, as this was a simulation of an actual clinical situation. The learner was expected to proceed according to what they had already learned and practiced. Reward systems were incorporated, with a maximum of 3 stars when the learner completed the procedures correctly. Deliberate practice and timely feedback on the learner’s performance were integrated into the case scenarios and situations to develop critical thinking and problem-solving abilities. Each IVR experience lasted 15-20 minutes to ensure no learner fatigue.

#### Implementation

**Figure 4:**
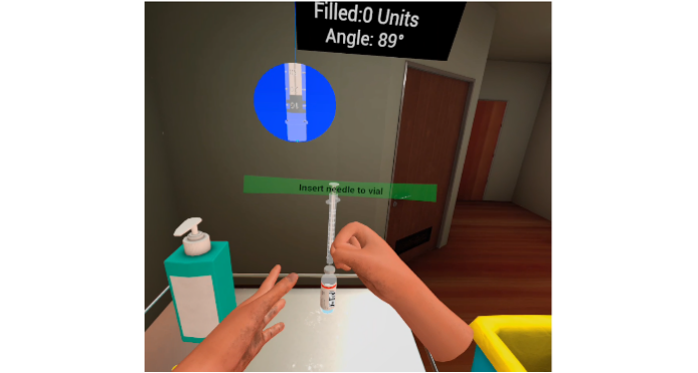
Subcutaneous injection.

**Figure 5:**
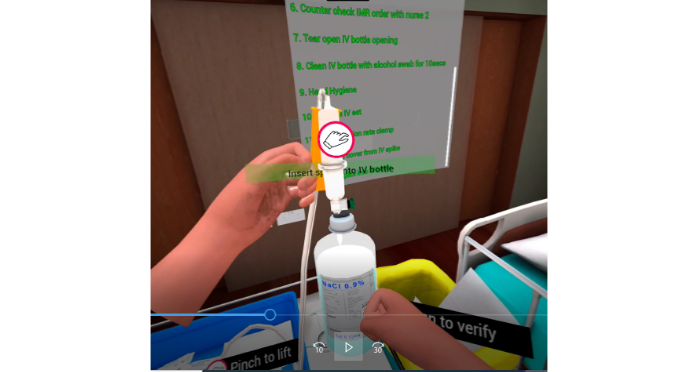
Intravenous therapy.

Figures 4 and 5 illustrate the subcutaneous injection and intravenous therapy procedures. In each scenario, the learners performed the procedures using the 2 handheld controllers. They also interacted with the patient and clinical instructor during critical steps such as checking identifiers and the “5 rights” when administering medication to ensure patient safety. The learners could click on a text box, and the patient would reply verbally with prerecorded responses. For example, if the learner asked, “May I know what your name and NRIC number are?” the virtual patient would reply, “My name is Ahmad, NRIC number is S1234567x.” An electronic scanner and medication record were created to provide realism for learners to check the patient and medication prior to the administration.

The virtual simulation of administering a subcutaneous injection (Figure 4) included the critical component of checking the correct instruments and materials, type of insulin, and techniques in withdrawing air prior to withdrawing the correct unit from the vial. The appropriate injection site and any complications were identified, such as pain, edema, erythema, temperature, and lipohypertrophy. Critical components, such as checking patient identifiers, hand hygiene, the correct 5 rights, and injection techniques, had to be performed correctly to pass the procedure.

For the intravenous therapy procedure (Figure 5), learners studied critical concepts such as using the correct fluids and the accurate rate of infusion when administering intravenous therapy. Safety aspects, such as maintaining aseptic techniques while spiking the drip set and connecting to the intravenous plug, and steps to prevent phlebitis were incorporated into the scenarios. The learners were also expected to examine the risk of air embolism by checking the intravenous tubing.

### Evaluation of IVR Clinical Procedure

#### Study Design

This usability study used a mixed methods approach using convenience sampling. The recruitment and intervention of participants took place from June to August 2021 upon receiving approval from the institutional ethics review board.

#### Participants

The participants comprised undergraduate nursing students in their second academic year who had completed the pathophysiology and pharmacology modules and the Nursing Practice 1 and Clinical Experience Practicum 1.2 modules.

#### Data Collection

To achieve the aims of the study, three questionnaires were administered to the participants, which collected the following information: (1) basic demographics and past VR experience, (2) perception of continuance intention, with an instrument developed by Roca et al [28], and (3) the System Usability Scale (SUS) developed by Brooke [29]. To maintain consistency, we explained how to use the head-mounted device and how to put it on to ensure it was in good working condition. Any device issues were rectified, and then the participants brought the IVR device home for 2 weeks of testing on their own time. Upon returning the device, they were invited to complete the questionnaires.

The scale for perception of intention to continue was adapted from Roca et al [28] and had the following subscales: (1) usefulness, (2) cognitive absorption, (3) ease of use, (4) system quality, (5) confirmation, (6) satisfaction, and (7) continuance intention. All items were rated on a 5-point scale (1=strongly disagree to 5=strongly agree), except for item 1 on the system quality subscale, which was scored in reverse. The subscales adapted in this study had Cronbach α values of .81 (usefulness), .94 (cognitive absorption), .93 (ease of use), .68 (system quality), .96 (confirmation), .96 (satisfaction), and .91 (continuance intention).

The SUS is a 10-item self-report measure investigating one’s reaction toward IVR procedures [29]. Items were assessed on a 5-point scale (1=strongly disagree to 5=strongly agree). The participants had to rate themselves on items like “I thought the VR procedure was easy to use.” For items 1, 3, 5, 7, and 9, the score is the scale position minus 1. For items 2, 4, 6, 8, and 10, the score is 5 minus the scale position. The scores for each item were summed and multiplied by 2.5 to calculate a total score ranging from 0 (lowest) to 100 (highest). There was no cutoff score, but higher total scores indicated higher levels of usability for the IVR procedures. The Cronbach α values for the scale ranged from .85 to .91 [30].

### Quantitative Analysis

Descriptive statistics using percentages, means, and SDs were computed to examine the study variables using SPSS (version 28.0; IBM Corp) [31].

### Qualitative Analysis

Qualitative data from the open-ended questions were imported into an Excel spreadsheet for coding. Content analyses were used to understand the users’ experiences of the IVR procedure [32]. Two researchers (STL and WLL) conducted the content analysis and extracted the codes, categories, and themes [33]. The research team reviewed the frequencies of the coded themes to reach a consensus. Trustworthiness, credibility, and transferability were ensured by including all 29 participants’ responses, selecting units of meaning, and presenting the findings in a rich and vigorous way (Multimedia Appendix 1).

### Ethics Approval

Ethical approval was obtained from the National University of Singapore Institutional Review Board (NUS-IRB 2021-305). The participants were briefed thoroughly, and the participant information sheet was provided prior to recruitment. Participants were informed that their participation was voluntary and that they could withdraw from the study at any time. Confidentiality and anonymity were ensured.

## Results

### Participants

In this study, 29 participants were recruited, and all completed the study. The mean age of the participants was 23.03 (SD 6.59) years, with the majority being female (n=18, 62%). There were 22 Chinese participants (76%), 3 Malay participants (10%), and 1 Indian (3%) participant, with 3 participants of other ethnicities (10%). Most had no experience using VR prior to the user testing (n=27, 93%).

### Quantitative Analysis

As presented in Table 1, for perception on intention, all the subscales in the perception scale had a mean score greater than 3 (of a total score of 5). This indicated that the students had a positive perception of the quality and usability of IVR, which is a key determinant for their continuity intention. The subscale with the highest mean score was “cognitive absorption” (mean 4.05, SD 0.61), suggesting that the students enjoyed and found the IVR engaging. The top 2 items with the highest scores supported this notion: “I have fun interacting with VR procedure” (mean 4.38, SD 0.68) and “When I am using the VR procedure, I am absorbed in what I am doing” (mean 4.14, SD 0.74). From another aspect, the 2 items with the lowest scores were “It is easy for me to become skillful at using VR procedure” (mean 3.21, SD 1.01) and “I will frequently use the VR procedure in future” (mean 3.24, SD 0.95).

However, for the student’s perception of the usability of IVR, the mean SUS score was 53.53 (SD 16.18). This score was below the acceptable score of 68, indicating that more work needed to be done to improve the usability of IVR. Two items with poor perception, that was, items with >10 participants, indicating “Do not agree for positively worded item” or “Agree for a negatively worded item,” were “I thought the VR procedure was easy to use” and “I think that I would need assistance to be able to use the VR procedure” (Table 2).

**Table 1:** Perception scale (N=29).

Subscales or items		Mean (SD)
**Usefulness**		3.71 (0.92)
	Using the VR^a^ procedure can improve my learning performance	3.79 (0.90)
	Using the VR procedure can increase my learning effectiveness	3.66 (0.97)
	I find the VR procedure to be useful to me	3.69 (1.11)
**Cognitive absorption**		4.05 (0.61)
	Time flies when I am using the VR procedure	4.00 (0.96)
	Most times when I get on to the VR procedure, I end up spending more time than I had planned	3.69 (0.81)
	When I am using the VR procedure, I am absorbed in what I am doing	4.14 (0.74)
	I have fun interacting with VR procedure	4.38 (0.68)
	I enjoy using the VR procedure	4.03 (0.82)
**Ease of use**		3.32 (0.86)
	Learning to operate the VR procedure is easy for me	3.31 (1.04)
	It is easy for me to become skillful at using VR procedure	3.21 (1.01)
	My interaction with the VR procedure is clear and understandable	3.45 (0.91)
**System quality**		3.50 (0.53)
	Number of steps per task is too many^b^	3.55 (0.63)
	Steps to complete the task follow a logical sequence	3.62 (0.86)
	The organization of information is clear	3.41 (0.98)
	The VR procedure has natural and predictable screen changes	3.55 (0.91)
	The VR procedure system is responsive	3.34 (1.01)
**Confirmation**		3.52 (0.84)
	My experience with using the VR procedure was better than I expected	3.48 (1.12)
	The service level provided by the VR procedure was better than I expected	3.55 (0.87)
	Overall, most of my expectations from using the VR procedure were confirmed	3.52 (0.78)
**Satisfaction**		3.70 (0.87)
	I am satisfied with the performance of VR procedure	3.31 (1.04)
	I am pleased with the experience of using the VR procedure	3.83 (0.89)
	My decision to use the VR procedure was a wise one	3.97 (0.98)
**Continuance intention**		3.46 (0.96)
	I will use the VR procedure on a regular basis in future	3.34 (1.08)
	I will frequently use the VR procedure in future	3.24 (0.95)
	I will strongly recommend others to use it	3.79 (1.11)
Overall score		3.63 (0.63)

^a^VR: virtual reality.

^b^Scored in reverse.

**Table 2:** System usability scale (N=29). The total score was 53.53 (SD 16.18).

Items		Strongly agree or agree, n (%)	Neutral, n (%)	Strongly disagree or disagree, n (%)
**Positively worded**				
	I think I would like to use VR^a^ procedure frequently	14 (48)	10 (34)	5 (17)
	I thought the VR procedure was easy to use	10 (34)	8 (28)	11 (38)
	I found the various functions of VR procedure were well integrated	12 (41)	10 (34)	7 (24)
	I would imagine that most people would learn to use VR procedure very quickly	11 (38)	10 (34)	8 (28)
	I felt very confident using the VR procedure	8 (28)	12 (41)	9 (31)
**Negatively worded**				
	I found the VR procedure unnecessarily complex	7 (24)	10 (34)	12 (41)
	I think that I would need assistance to be able to use the VR procedure	13 (45)	6 (21)	10 (34)
	I thought there were too much inconsistency on the VR procedure	6 (21)	7 (24)	16 (55)
	I found VR procedure very cumbersome or awkward to use	9 (31)	8 (28)	12 (41)
	I needed to learn a lot of things before I could get going with the VR procedure	8 (28)	9 (31)	12 (41)

^a^VR: virtual reality.

### Qualitative Analysis

#### Overview

Key themes were derived from the content analysis of the open-ended questions on the questionnaires regarding the experience of using VR procedures for learning. The themes derived from the analyses included the usefulness of IVR, learning experiences using IVR, challenges encountered, technical glitches, and suggestions for improvement. Themes and frequencies from the content analysis can be found in Multimedia Appendix 1.

#### Usefulness of IVR

Participants were excited about the new experience of using IVR. They found that the realism and relevance to the clinical setting and the ability to interact with a virtual patient and clinical environment promoted immersion in experiential learning:

It is intriguing as a first-time user; hence it caught my attention from the start. I could practice the nursing procedures at my own comfortable pace and get back to practicing the procedures again on my own time. There was an improvement in terms of accuracy in the procedure as I practiced more often on my own. Having a virtual patient in front of our eyes helped mimic clinical settings.

P11

Participants appreciated this learning format and found it a useful resource for students to practice and learn at their own pace, time, and convenience. “I can see this being a very useful resource for students to practice and learn in their own time” (P15).

#### Learning Experiences

Participants reported that performing the procedure using IVR encouraged them to consciously revise the sequence for the procedure and ensure accuracy as they built on their knowledge and skill through practice. This experience enhanced their thinking skills as they reviewed their performance:

I liked that the VR prompted me to think through the sequence of steps I have to do before a procedure. It helped me revise the steps and rationale behind the steps. I also like that the VR tracks your activity and lets you know which step you have forgotten to perform.

P3

Some participants commented that learning on the VR platform allowed novice learners to correct mistakes without putting an actual patient at risk:

The fact that I can practice clinical skills anywhere is an advantage of VR. Also, I would not be causing any real patient harm if I tried something out for the first time.

P23

It will make me understand my mistakes there, so I will be more aware not to repeat them the next try or during real-life clinical procedures.

P11

In particular, the participants appreciated the important concepts that might be overlooked in real clinical settings. One of these aspects was performing the “5 moments of hand hygiene” for any patient encounter. With IVR, the participants could not proceed, and they could see the “dirt” visually on their hands. “The visualization of soiled hands was also very useful as I see the importance of hand hygiene” (P21).

#### Challenges Encountered

Participants reported some limitations of the IVR system. The participants shared that adapting to the device and controls took time. Some concerns included physical discomfort, such as feeling dizzy, and that participants could not complete the entire procedure in 1 sitting:

I could not complete the tasks because I felt dizzy after a certain amount of time, and though I do revise the steps before I put on the headgear to do the task.

P27

Some of them also commented on their unfamiliarity with the device and instructions. Moreover, others were unable to communicate with the virtual patient and had a decreased level of enjoyment in using IVR:

I feel like there can be clearer instructions, possibly step by step, during my first attempt of the procedure to refresh and familiarize myself with the items. I could not communicate with the patient, grasp objects, put on gloves, and check the patient’s body.

P18

#### Technical Glitches

The most technical glitches involved the withdrawal and administration of the medication procedure. The participants shared that they experienced irritation when virtual items were not responding according to commands despite multiple attempts. This impeded their progress and learning as they needed to restart the procedures:

Some IT interface needs to be adjusted to smoothen the flow of procedural execution. Sometimes there is a glitch where the packaging of the alcohol swabs, needle, and IV line gets stuck to our hand, thus hindering our next steps.

P26

It was very frustrating that I could not inject the needle into the insulin vial and manipulate it too.

P22

#### Suggestion for Improvement

Three main areas for improvement were suggested: provide orientation and more specific instructions, include more audio and haptic feedback in the game, and optimize the controls and joystick. Some other suggestions included adding a progress bar to motivate students to practice, including more procedures, and extending the use to hospitals:

More instructions can be given. A good example would be IV priming, where additional instructions are taught to us.

P5

Please include more procedures...look forward to it. If it works well, this can be a good technology to bring into hospitals for staff re-education or enrichment purposes. And any updates to a particular nursing skill can be updated in the software as well...that will be excellent.

P6

## Discussion

### Overview

The objectives of this study were to describe the development and to evaluate the learning experience of undergraduate nursing students. Creating these 2 clinical procedures in VR involved multiple stakeholders, including multiple reviews and refinements prior to this study. A new teaching approach was incorporated to create an effective learning environment to promote competencies among nurses. This study was a timely development during the period of the pandemic, when there were movement restrictions, safe distancing, and physical segregation of staff and students. More essentially, this study met the learning needs of the next generation.

### Principal Findings

This study indicated that undergraduate nursing students found IVR satisfying and most of them enjoyed the experience. They were most intrigued by the contextualization of the VR procedures and felt that the experience was better than they had expected. This result reflects findings in the broader literature [34]. Once in the VR environment, they were absorbed in performing the procedures and tended to spend more time than intended. One of the reasons could be the high degree of realism, as the VR environment was a close representation of the actual ward setting. The participants could interact with patients when guided by the clinical instructor, just like in real life. From the study, the scores in the perception scale and qualitative data showed that the participants were highly engaged and immersed and forgot the time when performing the VR procedures, which is congruent with other studies [35].

Some participants shared that when practicing at home with practice versions of necessary instruments and materials, they were only able to practice psychomotor skills and that there was no holistic patient experience. They liked the VR system as it simulated the actual ward with instruments and materials readily available. The high-resolution visuals were stimulating for the students as they performed the task. In addition, the VR procedures were rolled out during the COVID-19 pandemic, when in-person practice sessions were largely reduced. The lack of hands-on practice experience might be an impetus for students to embrace using technology for learning more readily. The students were keen to experience a new approach to learning and viewed it as a valuable learning opportunity.

In this study, most participants agreed that VR effectively allowed them to practice steps, enabled repeatable learning, and helped them form good habits, including hand hygiene habits, such as when they touched a patient or the environment with their hands and prior to performing any procedures. They were able to remain focused without being distracted by the external environment. In addition, IVR allowed the participants to proceed at their own pace and enabled them to construct new knowledge through the reflective thinking process. With the checklist embedded in the VR procedures, the students could receive timely feedback during their practice. Moreover, they had a sense of achievement when they completed the procedures successfully. The real-time feedback motivated the participants, and they strived to do better in each practice session. Such a feature is critical to enhancing learning, as Generation Z students look for instant results [4,5]. Most participants agreed that VR provided a good learning platform and that it helped them to be more focused on finer details to improve their competencies. The interactive clinical procedures were valuable as they encouraged self-regulated learning. The learners were able to develop new knowledge linked to previous learning when they eventually progressed from virtual practice to actual practice. This explains the willingness of undergraduate students to explore the IVR system, as it provided opportunities for them to put theory into practice and could improve their confidence in real-life practice [34].

### Comparison to Prior Work

These findings are aligned with other studies showing that IVR is promising for improving engagement and motivating students to learn. Hence, this learning platform is suitable to be included as a teaching approach [36,37]. However, in this study, the ease of use and system quality of the VR procedures scored lower than the rest of the perception subscales. Similarly, the SUS also showed that more than half of the participants felt they required assistance to use the VR system, and that it was not easy to use. This signifies the importance of improving the efficiency and quality of the VR prototypes. The participants shared in their qualitative feedback that the controls were not intuitive and were confusing, as they were unsure which button to click. In addition, they reported technical issues, such as that the items kept sticking to their hands or dropping. This feedback helped the IT staff improve the procedures before implementing them for the entire cohort of students.

The participants expressed that they needed time to get used to and learn to use the VR device. Some used the VR set for a prolonged period, resulting in physical discomfort and cybersickness. This study highlighted that not all users find IVR comfortable, and that more refinement needs to be made to reduce technical difficulties. Therefore, IVR might not be suitable for everyone [38]. To ensure cyber safety, more precise tutorials and instructions should be developed to guide users. The user guide for the VR technology and the controllers will be revised. Maximum play duration and cautions and instructions for safety must be included in the orientation for users.

Finally, the participants also found the application lacking in audio and haptic feedback. They suggested including more cues by having vibration or buzzing when errors are made or having a visual dialogue box or audio prompt to inform players to increase interactivity. They shared that students would learn more if there were more challenging situations, such as including several types of intravenous solutions or insulins to stimulate students’ thinking. These ideas were valuable feedback for enhancing the learning experience, and the team has considered refining the prototypes.

### Strengths and Limitations

The strengths of this study included, first, that all interactive clinical scenarios were reviewed and refined by the various parties. Second, faculty and students conducted several rounds of user acceptability testing to refine the prototypes prior to this study. Finally, developing the clinical procedures was a collaborative effort among clinical, academic, and IT professionals. All comments received were valuable, and improvements suggested by the participants were incorporated into the future refinement of the IVR procedures. The limitations included the IT professionals using Unreal Engine software for the first time, which led to teething issues. As they gained expertise in using the program, the IVR procedures significantly improved. Although the sample size was small, considering that it was a usability study, 29 participants was considered a sufficient size, and data collection for reviewing the design and evaluating its usefulness reached saturation. Going forward, more participants will be recruited to test the effectiveness of the IVR procedures.

### Conclusions

IVR for simulating intravenous therapy and subcutaneous injection procedures was designed and developed for nursing students to perform the procedures at their own convenience and the time and place of their choosing. Our evaluation supports the feasibility of using IVR in learning clinical procedures. It highlighted the value of deliberate practice and scaffold learning via VR procedures. The study also supported the usability of the VR device, although improvements and revisions are needed for a better user experience. With the enhanced features, future research can examine the effectiveness of health care students’ learning clinical procedures at home using IVR. Given its portability and flexibility, IVR is potentially an effective adjunct to support health care students in learning and performing clinical procedures.

## Appendix

Multimedia Appendix 1 Content analysis.

## Abbreviations

IVR: immersive virtual reality
SUS: system usability scale
VR: virtual reality

## Data Availability

The data sets generated and analyzed during this study are not publicly available for privacy and ethical reasons but are available from the corresponding author upon reasonable request.
