# Supplementary material for: Design and Evaluation of Using Head-Mounted Virtual Reality for Learning Clinical Procedures: Mixed Methods Study
Source: JMIR Serious Games. 2023 Aug 30;11:e46398. doi: 10.2196/46398 (PMC10500353; doi:10.2196/46398)
Supplement: Multimedia Appendix 1 [file games_v11i1e46398_app1.docx]

Content Analysis

| **Category description** | **Frequencies** | **Percentage** |
| --- | --- | --- |
|  |  |  |
| **Usefulness of the IVR** | 34 |  |
| Working at own time/pace | 5 | 14.71 |
| Clear instructions/checklist | 4 | 11.76 |
| Provides feedback/prompts | 4 | 11.76 |
| Redo procedures/revise steps | 3 | 8.82 |
| Display of requisites | 3 | 8.82 |
| Easy to use | 2 | 5.88 |
| Improvements to accuracy of procedure | 2 | 5.88 |
| Alternative to hands on practice | 2 | 5.88 |
| Useful tool for students | 2 | 5.88 |
| Use for education/assessment purposes | 2 | 5.88 |
| Activity tracking | 1 | 2.94 |
| Less stress than labs | 1 | 2.94 |
| Cannot replace hands on practice | 1 | 2.94 |
| Adjunct to labs | 1 | 2.94 |
| Easier to update nursing skills | 1 | 2.94 |
| **Learning experiences** | 45 |  |
| Realistic/immersive | 11 | 24.44 |
| Interactive/engaging/fun/cool | 9 | 20.00 |
| Novel/first experience | 7 | 15.56 |
| Pleasant experience | 6 | 13.33 |
| Simulation of real life | 3 | 6.67 |
| Takes time to get used to IVR | 3 | 6.67 |
| Cannot track progress | 2 | 4.44 |
| Objective viewed upon completion | 1 | 2.22 |
| Unpleasant experience | 1 | 2.22 |
| Procedures more complicated than real-life | 1 | 2.22 |
| Like actions for hand hygiene | 1 | 2.22 |
| **Challenges encountered** | 50 |  |
| Physical discomfort | 8 | 16.00 |
| Unable to complete tasks/actions | 8 | 16.00 |
| Inconsistent visuals/actions/controllers | 8 | 16.00 |
| Actions not intuitive with controllers | 8 | 16.00 |
| Interacting with patient | 4 | 8.00 |
| Sensitivity needs improvement | 4 | 8.00 |
| Cannot perform fine motor skills | 4 | 8.00 |
| Failed to capture user's response | 2 | 4.00 |
| Not interactive | 1 | 2.00 |
| NPC not helpful | 1 | 2.00 |
| Adjust flow of procedural execution | 1 | 2.00 |
| Mistakes not reflected in checklist | 1 | 2.00 |
| **Technical glitches** | 45 |  |
| Withdrawing/administering medication | 11 | 24.44 |
| VR gloves for better control | 4 | 8.89 |
| Items to snap properly | 4 | 8.89 |
| Need to restart/troubleshoot | 3 | 6.67 |
| Items dropping/disappearing | 3 | 6.67 |
| Priming IV line | 3 | 6.67 |
| Sanitising hands | 3 | 6.67 |
| Removing packaging | 3 | 6.67 |
| Grasping objects | 2 | 4.44 |
| Flicking air bubbles | 2 | 4.44 |
| Maintaining aseptic technique | 1 | 2.22 |
| Scooping insulin needle | 1 | 2.22 |
| Moving requisites to bedside | 1 | 2.22 |
| Items not respawning | 1 | 2.22 |
| Needles falling through sharps box | 1 | 2.22 |
| Items overlapping each other | 1 | 2.22 |
| App needs to be streamlined | 1 | 2.22 |
| **Suggestion for improvement** | 54 |  |
| More specific instructions/orientation | 12 | 22.22 |
| Audio/haptic feedback in game | 12 | 22.22 |
| Optimisation of controls/joystick | 6 | 11.11 |
| Clearer font/learning objectives | 3 | 5.56 |
| Space constraints | 2 | 3.70 |
| More user friendly/interactive | 2 | 3.70 |
| Update on wrong/missing steps | 2 | 3.70 |
| Ensure new users’ complete tutorial | 2 | 3.70 |
| Different patient name from orders | 2 | 3.70 |
| Demo version | 2 | 3.70 |
| Highlight cleaned area after swab | 1 | 1.85 |
| Provision of charging adapter | 1 | 1.85 |
| Redo/undo steps | 1 | 1.85 |
| Have a progress bar | 1 | 1.85 |
| Have more procedures | 1 | 1.85 |
| Loan to all nursing students | 1 | 1.85 |
| Improve visuals | 1 | 1.85 |
| Varying levels of difficulty | 1 | 1.85 |
| Respawning of items | 1 | 1.85 |
